# Event-based prospective memory in patients with Parkinson’s disease: the effect of emotional valence

**DOI:** 10.3389/fnhum.2015.00427

**Published:** 2015-07-24

**Authors:** G. Mioni, L. Meligrana, P. G. Rendell, L. Bartolomei, F. Perini, F. Stablum

**Affiliations:** ^1^Department of General Psychology, University of PadovaPadova, Italy; ^2^U.O. Neurologia, Ospedale San BortoloVicenza, Italy; ^3^U.O. Psicologia Ospedaliera, Ospedale San BortoloVicenza, Italy; ^4^School of Psychology, Australian Catholic UniversityMelbourne, VIC, Australia

**Keywords:** prospective memory, event-based, Parkinson’s disease patients, emotional valence, Virtual Week

## Abstract

The present study investigated the effect of Parkinson’s disease (PD) on prospective memory (PM) tasks by varying the emotional content of the PM actions. Twenty-one older adults with PD and 25 healthy older adults took part in the present study. Participants performed three virtual days in the Virtual Week task. On each virtual day, participants performed actions with positive, negative or neutral content. Immediately following each virtual day, participants completed a recognition task to assess their retrospective memory for the various PM tasks. PD patients were less accurate than the control group at both PM accuracy and recognition task accuracy. The effect of emotional valence was also evident, indicating that all participants were more accurate on positive PM tasks than both negative and neutral. This study confirmed PM impairment in PD patients and extended previous research showing how positive emotional stimuli can influence PM performance.

## Introduction

Prospective memory (PM) refers to memory for future intentions and involves remembering to perform an action in response to a specific cue, while being involved in an ongoing activity (McDaniel and Einstein, 2007; Kliegel et al., 2008b). In order to successfully complete PM actions, participants are required to remember the content (retrospective component) of the PM action and perform it in the future (prospective component); either at a set time (time-based PM) or when an appropriate cue occurs (event-based PM; Einstein and McDaniel, 1990). Cue detection has been identified as one key aspect in modulating PM performance; in fact, highly salient cues facilitate a relatively automatic focus of attention towards the cue and therefore decrease the need for intentional cue detection (McDaniel and Einstein, 2000). Cue manipulation in PM paradigms have mainly been done in terms of cue familiarity (McDaniel and Einstein, 1993, 2000; Brandimonte and Passolunghi, 1994) or focality (Kliegel et al., 2008a; Rose et al., 2010). Interestingly, it has also been observed that PM cues with emotional valence are better remembered than PM cues with non-emotional content. The distinctiveness of an emotional cue may reduce the need for controlled monitoring of the cue as its detection is facilitated, and this may result in better PM performance (Kliegel and Jäger, 2006; Murphy and Isaacowitz, 2008).

For example, when the PM task included words Clark-Foos et al. (2009) found that PM cues (embedded in a lexical decision task) with positive emotional valence were detected more often than PM cues with negative emotional valence in young adults. Schnitzspahn et al. (2012) used a color-matching task in which participants saw a series of colored rectangles displayed one by one followed by a word. Participants indicated whether or not the color of the word matched the color of the rectangles. Five target words within a block had emotional valence. Results showed that age differences were smaller in both positive and negative emotional valence conditions. For older adults, positive cues improved the prospective component of the PM action, while negative cues improved the retrospective component. No main effect of valence was found for younger adults (Schnitzspahn et al., 2012). Finally, May et al. (2012, 2015) in two experimental studies asked younger and older adults to make word/non-word judgments and the valence of the PM targets varied across blocks. Results showed that positive and negative PM cues led to a similar improvement of PM performance as compared to neutral PM cues. The authors’ interpretation of the results indicate the efficacy of emotion in boosting cue saliency, reducing the need for strategic monitoring (May et al., 2012) and also that older adults can effectively use emotional cues to help them initiate actions and to minimize repetition errors (May et al., 2015).

Similar emotional enhancement in PM performance was also observed when the PM emotional stimuli included images. For example, Altgassen et al. (2010) included both younger and older adults that were tested with a one-back visual working memory paradigm (ongoing task) that included neutral, positive, and negative pictures (pictures were taken from the International Affective Picture System; Lang et al., 2005). The PM task required participants to press a different key when the PM cues appeared. Emotional enhancement (both positive and negative) was observed and age-related deficit was observed only when neutral PM cues were presented. Also, Rendell et al. (2011) tested younger and older participants with Virtual Week (Rendell and Craik, 2000) and manipulated the pictures assigned to the PM tasks to have positive, negative or neutral content. The authors observed a positive enhancement compared to neutral PM cues in both age groups but no effect of negative emotional stimuli on PM performance. Overall, older adults were less accurate than younger participants, but benefited more greatly from positive valence stimuli than young adults.

Taken together, it seems clear that stimuli with emotional valence increased PM performance with a greater enhancement of stimuli with positive emotional valence (Kliegel and Jäger, 2006; Murphy and Isaacowitz, 2008; Clark-Foos et al., 2009; Altgassen et al., 2010; Rendell et al., 2011; Schnitzspahn et al., 2012). While the extant studies are suggestive, additional data is needed for a more complete understanding of the interplay between PM and emotion processing in clinical populations. In fact, PM is involved in many real-world tasks, such as remembering to take medication and to attend appointments, and it is a crucial process for maintaining healthy and safe independent living. Moreover, investigating the effects of emotional valence on PM performance are also of particular interest because everyday intentions are often not simply neutral tasks but are associated with emotional information (e.g., calling a good friend for his/her birthday or buying medicine at the pharmacy for an ill friend). Finally, specifically for clinical populations, investigating possible emotional enhancement in patients’ PM performance could have important implications for rehabilitation programs.

As far as we know, only two studies have been conducted to investigate emotional enhancement in clinical populations. Rendell et al. (2012) used Virtual Week and tested patients with multiple sclerosis and healthy matched controls. Patients performed worse than the control group in both event- and time-based PM tasks, but a positive enhancement was observed only in event-based tasks in both groups. Again, positive enhancement was observed by Altgassen et al. (2011) in event-based PM tasks (word categorization task) conducted with depressed patients and healthy controls. Healthy adults outperformed individuals with depression in the PM task. Depressed patients and healthy controls differed when responding to PM cues with positive valence, reflecting a positive enhancement only in healthy adults. In the case of depressed patients, there was a trend for better performance on the neutral than on both the positive and negatively valenced cues. This suggests that for this specific population, emotional salience does not generate benefits in PM performance (Altgassen et al., 2011; Stuhrmann et al., 2011).

In recent years, an increasing interest in PM performance in Parkinson’s disease (PD) patients has been observed. PD patients frequently report cognitive decline in various executive function domains such as: inhibition, switching, and planning (Lewis et al., 2003; Woods and Tröster, 2003; Muslimovic et al., 2005). Cognitive deficits have also been reported in working memory or attentional tasks (Lewis et al., 2003; Muslimovic et al., 2005). Finally, PD patients showed some retrospective memory dysfunction in particular, they showed impaired performance on free recall tasks, but spared performance on recognition and cued recall tasks (Whittington et al., 2000, 2006). The early occurrence of deficits involving executive functions in PD patients has been attributed to the depletion of dopamine at the level of both basal ganglia and prefrontal cortex, which is responsible for the dysfunction of the prefrontal-striatal pathways in these persons (Owen, 2004; Cools, 2006).

Considering that PM is a multiphase and complex construct that relies on integrity of executive functions (i.e., planning forming and executing the intention, monitoring for the appropriate moment to initiate the intended action, inhibit the ongoing activities and switch from ongoing activities to perform the intended action; McDaniel and Einstein, 2000; Kliegel et al., 2011) and that most of these cognitive functions rely on the functional integrity of the frontal systems (Glisky, 1996; Martin et al., 2003; McFarland and Glisky, 2009) PM dysfunctions in PD patients should be expected.

For example, Kliegel et al. (2005) used a complex PM paradigm to test PM dysfunction in intention formation, intention retention, intention initiation, and intention execution of PD patients and tested for the mediating effect of executive functions. The authors found that PD patients were selectively impaired in forming and initiating the detailed intentions. Since both PM stages are representative of executive functions like planning and inhibition, failure in these steps suggests poor executive functioning in PD patients (Kliegel et al., 2011).

Considering the studies that used event- and time-based tasks, PD patients were less accurate than controls in both PM tasks (Ramanan and Kumar, 2013). Interestingly, PM performance in PD patients was as accurate as controls when more importance was dedicated to the PM task compared to the ongoing task, indicating that PM impairment in PD patients seems to be mainly caused by reduced working memory abilities to process together the PM and the ongoing task (Altgassen et al., 2007). Moreover, varying the strategic (non-focal condition) or the spontaneous (focal condition) retrieval,^1^ PD patients were less accurate than controls in the non-focal but not in the focal condition. These confirmed that PD patients were preferentially impaired on PM tasks for which higher levels of executive control were needed to support intention retrieval (Foster et al., 2009). Finally, previous studies reported PM impairment in PD patients despite the fact that they generally remembered the content of the PM actions (Katai et al., 2003; Kliegel et al., 2005; Costa et al., 2008a; Foster et al., 2009). This suggests that the retrospective memory processes involved in encoding and retaining the intention content are intact, while the executive processes underlying self-initiated intention retrieval or execution at the appropriate moment in the future are impaired (prospective component). However, different results were also observed. Costa et al. (2008a), in fact, showed that PD patients were less accurate than controls in recalling the specific actions to be performed in both the time- and event-based tasks, and they were as accurate as controls when performing event-based tasks.

Methodological differences between these studies may explain the different results; but also the notion that particular features of PM tasks can influence PM performance has begun to guide more refined experimental studies (McDaniel and Einstein, 2000). Foster et al. (2009) manipulated cue-focality and found that while PD participants were impaired on tasks with non-focal cues, they were as accurate as the control group on tasks with focal cues. Foster et al. (2013) using Virtual Week (Rendell and Craik, 2000) extended these findings and manipulated cognitive demand (focal vs. less focal cues) and retrospective memory demand (regular vs. irregular PM tasks). For irregular PM tasks, PD patients were less accurate than the control group regardless of the type of cue (focal or less focal), whereas for regular PM tasks, PD patients were less accurate than the control group only when the cue was less focal. These studies suggested that PM in PD patients can be supported by cue-related features that facilitate automatic intention retrieval (Kliegel et al., 2008a; Foster et al., 2009; Rose et al., 2010).

Interestingly, as previously introduced, Altgassen et al. (2007) tested the hypothesis that varying the task importance during the intention formation phase might improve PM performance and they tested this hypothesis with PD patients and controls in an event-based task. The rationale for this assumption rests on studies which have shown PM improvement in tasks that were perceived as highly important (Kliegel et al., 2001). The task which is perceived as more important receives more attention and consequently, performance on this task is enhanced (Kliegel et al., 2001, 2004). The results showed that PD patients can be as accurate as the control group when they concentrate on the PM task, suggesting that PM performance can be improved even in PD patients. These findings have important implications from a clinical point of view, suggesting that PD patients with reduced executive functions (Lewis et al., 2003; Muslimovic et al., 2005; Whittington et al., 2006) may perform PM tasks as accurately as the control group if the correct conditions exist.

Stimuli with emotional valence can be better and easily detected and can boost PM performance (Kliegel and Jäger, 2006; Murphy and Isaacowitz, 2008; Clark-Foos et al., 2009; Altgassen et al., 2010; Rendell et al., 2011; Schnitzspahn et al., 2012); moreover, the effects of emotionally valenced cues on PM performance is of particular interest because everyday activities are often associated with emotional information and not only neutral tasks. Therefore, is it possible to boost PM performance in PD patients by using emotionally valenced stimuli?

So far, no study has explored the influence of emotional cues on PM performance in PD patients. To investigate the possible benefits of emotional cues on PM performance the content of the PM actions in the intention formation phase were manipulated. In particular, actions with positive, negative and neutral content were selected and assigned each action to an associated image with the same emotional valence (i.e., “Pay a speeding fine” was associated with an image showing a person given a speeding fine by a police officer). Following previous findings by Rendell et al. (2012) with multiple sclerosis patients, it was decided that the emotional valence of the PM actions will only be manipulated on event-based PM tasks and therefore it will exclude time-based tasks. In event-based tasks participants respond to the target cues that trigger the PM actions while time-based tasks are guided by self-monitoring behavior and are not related to a presentation of a cue, therefore, the association between task content and task cue can be stronger in event-based tasks.

The present study had two main aims. Firstly, the investigation of PM performance in PD patients using a computerized task that simulated every day activities. Consistent with previous findings, PM dysfunctions in PD patients was predicted. In particular, the present study aimed to investigate if the PM dysfunction observed in PD patients is mainly caused by a dysfunction at the prospective or retrospective memory component of PM process. To this end, a recognition test of the PM content was included at end of each virtual day. According to the literature, we predicted that PM dysfunction in PD patients is mainly caused by a dysfunction at the PM component rather than at the retrospective memory component. Secondly, the effects of emotional cues on PM performance were investigated and the emotional content of the PM task was manipulated at encoding with expectation of an emotionally related improvement in PM performance. More precisely, we investigated whether PM actions with emotional valence will be better encoded and remembered compared to PM actions with neutral valence, in particular we predicted a positive enhancement. It was further tested whether the presentation of emotional images will interact with group (PD, controls), possibly attenuating PD-related PM dysfunctions.

## Materials and Methods

### Participants

Twenty-one older adults with PD (*M* = 9, *F* = 12) and 25 healthy older adults (*M* = 10, *F* = 15) took part in the present study. PD participants were recruited from the Department of Neurology at the Hospital of San Bortolo, Vicenza, Italy and non-PD participants were volunteers from the local community. Table 1 reports demographic and clinical characteristics of the two groups. All PD participants had been diagnosed with idiopathic PD by a movement disorder neurologist and were between 1 and 17 at “Unified Parkinson’s Disease Rating Scale” (UPDRS); Fahn and Elton, 1987) which evaluates the progression of a person’s PD. Exclusion criteria for the PD group and the Control group included possible dementia or global cognitive impairment (Mini-Mental State Examination, MMSE, score <24; Folstein et al., 1975) and treatment with anticholinergic medications, treatment with certain dopaminergic or benzodiazepine medications known to interfere with cognitive functioning, history of neurosurgery or other neurological conditions (aside from PD for PD participants), history or current psychiatric disorder, or any condition which would interfere with testing. No patients showed an “on-off” phenomenon.

**Table 1:** **Mean (M) and standard deviation (*SD)* for the characteristics of PD patients and controls**.

	PD group *n* = 21	Control group *n* = 25
	*M (SD)*	*M (SD)*	*t*	*d*
Age (years)	68.95 (5.38)	71.12 (6.37)	1.23	0.36
Education (years)	7.76 (3.90)	7.88 (3.05)	0.11	0.03
MMSE	27.28 (1.85)	28.00 (.87)	1.74	0.49
FAB	15.95 (1.71)	16.32 (1.14)	0.87	0.25
BDI	3.53 (3.69)	2.92 (7.21)	0.33	0.10
Years of disease duration	5.7 (4.33)			
UPDRS (on stable medication)	10.92 (5.55)	–	–	–
NPI	4.75 (6.75)	–	–	–

*Note: MMSE, Mini Mental State Examination; FAB, Frontal Assessment Battery; BDI, Beck Depression Inventory; UPDRS, Unified Parkinson’s Disease Rating Scale and NPI, Neuropsychiatric Inventory*.

The *Frontal Assessment Battery* (FAB; Appollonio et al., 2005) was used to evaluate frontal lobe function and thus being able to identify a dysexecutive syndrome (higher score 18). The *Beck Depression Inventory* (BDI) self-report questionnaire (Beck et al., 1961) was assessed to evaluate the level of depression in PD patients and to evaluate the risk of depression in controls.

The two groups did not differ significantly with respect to age, years of education, MMSE, FAB and BDI (*p*s > 0.05; Table 1 reports the *t* values and Cohen’s *d*).

*Neuropsychiatric Inventory* (NPI; Cummings et al., 1994) was also assessed in the PD group for evaluating psychopathology. Controls were recruited from community of Vicenza and Bari (Italy) and were matched to the clinical participants on the basis of age and years of education (±2 years with respect to PD sample).

### Procedure

Participants were tested individually during two sessions that lasted approximately 90 min each: during the first session participants performed the neuropsychological tasks while during the second session participants performed the Virtual Week task. PD participants were tested while on their regular anti-PD medications. Demographic information for both groups was obtained through interviews and PD-related clinical characteristics were obtained from clinical chart reviews. Informed consent was collected from all participants and the study was conducted in accordance with Helsinki Declaration (59th WMA General Assembly, Seoul, 2008) and the guidelines of Department of General Psychology (Padova, Italy).

#### Prospective Memory Test: Computerized Virtual Week

The present study used a computerized version of Virtual Week (Rendell and Henry, 2009). Virtual Week is a laboratory measure of PM designed to represent PM in daily life. It represents a computer board game, in which participants move around the board with the roll of a die. Each circuit of the board represents a virtual day with things to do and decision to make. At the beginning of each virtual day participants received instruction about two actions to remember performing during the virtual day. Two additional tasks were given during the day (total four activities each day). Participants did not physically execute the task, but when the appropriate cue appeared they were instructed to click on the “Perform Task” button and select from the list the action required. The version used in the present study is an adaptation of the original version translated into Italian (for a more detailed description of the Virtual Week, see Rendell and Craik, 2000; Mioni et al., 2013, 2015). Importantly, in the present version participants are required to execute three virtual days (from Monday to Wednesday) and were required to perform four irregular event-based PM tasks every virtual day. Irregular activities vary every virtual day; moreover, we decided to include only event-based tasks based on previous studies that did not find emotional enhancement on time-based tasks using Virtual Week (Rendell et al., 2011, 2012).

Each event-based task was presented with a task relevant photo from the International Affective Picture System (IAPS; Lang et al., 2005). Each IAPS picture has unique standardized values, based on 1–9 rating scale of valence (unpleasant to pleasant) and arousal (calm to excited). Three sets of four emotional pictures were selected to constitute positive (mean valence = 7.62, *SD* = 1.62; mean arousal = 5.42, *SD* = 2.36), negative (mean valence = 2.65, *SD* = 1.69; mean arousal = 4.75, *SD* = 2.22) and neutral (mean valence = 5.03, *SD* = 1.46; mean arousal = 2.86, *SD* = 2.41) images based on the IAPS standardization. The event-based tasks were modified from earlier versions of Virtual Week so that they would have positive, negative or neutral content that corresponded to a specific IAPS image. Examples of positive, negative or neutral activities are: “Tell Roberta that Maria had a baby girl when you talk to Roberta”; “Pay a speeding fine when you go shopping” and “Buy the bus tickets after breakfast”. Specifically for Virtual Week, the PM cue is the title that appears in the Event card; so, with respect to the examples provided the PM cues are: “talking to Roberta”, “shopping” and “after breakfast”. A practice day was included before the experimental session, consistent with traditional administrations of Virtual Week. During the trial day participants performed 4 event-based activities with neutral emotional valence. During the experimental phase, participants were required to complete 12 event-based tasks (four positive, four negative and four neutral). Each day included two tasks of one valence and one of each of the other two valence categories (presented in counterbalanced order; see Rendell et al., 2012 for similar procedure). Participants performed each virtual day in about 15–20 min, breaks were included between days.

#### Recognition Test of PM Task Content

Immediately following each virtual day, participants completed a recognition test to assess their retrospective memory for the various PM tasks. The test required matching each intended action with its cue (i.e., PM action = “Pay a speeding fine”; PM cue = “shopping”). Participants were presented with a list of eight actions (e.g., pick up dry-cleaning), four of them were required during the virtual day and four were distracters. For each task, participants selected the matching cue from a pull down menu, listing the cues (e.g., when shopping, at university). The list of cues includes “not required” as one of the options to select, which is the correct response for the distractors that were included in the list of actions. Proportion correct was calculated for each emotional valence task (positive, negative and neutral). We considered false alarm when the participant recognized a distractor as a PM actions included in the virtual day.

#### Neuropsychological Evaluation

Participants also performed a battery of neuropsychological tests that evaluate non-verbal intelligence, executive and memory functions with reference to normative data in the Italian population.

*Colored Progressive Matrices* (CPM; Carlesimo et al., 1996): are made up of a series of designs with a part missing. Patients taking the tests are expected to select the correct part to complete the designs from a number of options printed beneath. The “colored” version is often used with patients with cognitive impairment and includes three different series of 12 items with increasing difficulties. The CPM evaluates cognitive functioning and non-verbal intelligence. It is scored by adding the correct responses at each item (higher score indicates better performance).

*Semantic Fluency* (Novelli et al., 1986): requires patients to generate words belonging to a category: fruit, cities, color, and animals. Each of the four trials lasts 60 s. It is scored by adding the number of words produced. Semantic fluency test is a good indicator of cognitive flexibility and lower performance might indicate cognitive impairment (Semenza, 1996). Higher score indicates better performance.

*Trial Making Test* (TMT; Giovagnoli et al., 1996): is composed of two parts (part A and part B). In part A, participants are required to connect a series of 25 numbers in numerical order. In part B, the subject connects 25 encircled numbers and letters in numerical and alphabetical order, alternating between the numbers and letters. Part A is generally presumed to be a test of visual search and motor speed skills; whereas part B is considered also to be a test of higher level cognitive skills such as mental flexibility. Performance is evaluated in time (seconds) to execute the task. In the present study we use the time to execute Part A and Part B and the difference in time to execute Part B and Part A (Part B–Part A). Higher scores indicate lower performance.

*Word list recall* (Carlesimo et al., 1996): consists of five consecutive immediate free-recall trials of a list of 15 words read aloud by the examiner (immediate recall; score range = 0–75). After 15 min, a delayed recall trial is given (delayed recall; score range = 0–15). Higher scores indicate better performance.

*Prose recall* (Mondini et al., 2003): The participant is asked to recall a short story read aloud by the examiner immediately after presentation (immediate recall), then the examiner reads the story again and asks to the participants to pay attention to the story because after that it is required to repeat it again. After a delay of 20 min interval, in which the participants are engaged in non-verbal tasks, the examiner asks to repeat the story (delayed recall). Higher scores indicate better performance.

### Statistical Analyses

PM participants’ performance was analyzed in terms of proportion of correct responses. This was the number of correct responses, expressed as proportion of the four PM tasks scheduled for each of the three categories of emotional cues: positive, negative, and neutral. Data were analyzed with a 2 × 3 mixed ANOVA with the between-subjects variable of *group* (Parkinson, controls) and the within-subjects variable of *emotional cue* (positive, negative, neutral). All significant effects were followed by *post hoc* analyses performed with a Bonferroni correction to reduce the Type I error rate, and the effect size was estimated with partial eta squared (${\text{η}}_{p}^{2}$).

To clarify the impact of emotional valence: positivity and negative enhancement/impairment indices were calculated as the difference between proportion correct on the positive minus the neutral tasks, and the negative minus the neutral tasks. This is consistent with Murphy and Isaacowitz (2008), who advocated comparing positively or negatively valence material with neutral material.

Participants’ performance on recognition test was also analyzed in terms of proportion of correct responses of the four PM tasks in each category: positive, negative, and neutral (see also Terrett et al., 2014 and Mioni et al., 2015 for similar procedure). The 2 × 3 mixed ANOVA conducted with proportion correct on PM task was replicated with the retrospective memory test. False alarms were possible and occurred when participants assigned a cue to a distraction action (actions not required during the virtual day). However, false alarms in the recognition task were too few to warrant consideration in analysis of accuracy on recognition task; a total of six and one false alarm/s for the entire PD and control groups respectively.

Separate *t*-test analyses were conducted between PD patients and controls to investigate the performance at neuropsychological tasks.

Correlation analyses were also conducted separately for PD patients and controls to investigate the relationship between PM accuracy and accuracy at the recognition task and to investigate the involvement of cognitive abilities and clinical measures on PM performance and performance at the recognitiontask.

## Results

### Prospective Memory Task: Analysis of Emotional Valence

**Figure 1:**
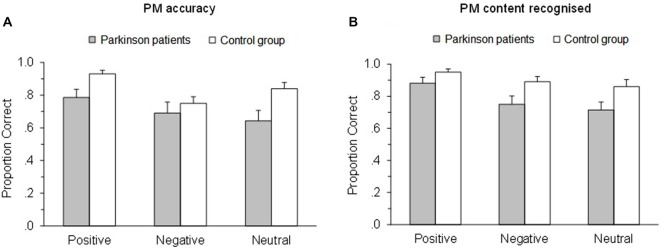
**Proportion of correct responses (A) and Proportion of correct responses on the retrospective memory test (B) as a function of emotional valence of the tasks and groups**. Error bars depict standard error of the mean.

Participants’ performance as function of the emotional valence of the PM tasks and groups are reported in Figure 1A and Table 2. Results showed that group did not interact with emotional cue (*p* = 0.214, ${\text{η}}_{p}^{2}$ = 0.03), but there was a main effect of group, *F*_(1,44)_ = 6.85, *p* = 0.012, ${\text{η}}_{p}^{2}$ = 0.14, with the people with Parkinson’s (*M* = 0.71, *SD* = 0.28) less accurate than controls (*M* = 0.84, *SD* = 0.19). There was also a main effect of emotional cue, *F*_(2,88)_ = 7.16, *p* = 0.001, ${\text{η}}_{p}^{2}$ = 0.14. *Post hoc* tests revealed that all participants were more accurate on positive PM tasks (*M* = 0.86, *SD* = 0.19) than both negative (*p* = 0.003, *M* = 0.72, *SD* = 0.25) and neutral (*p* = 0.006, *M* = 0.75, *SD* = 0.26), but participants did not differ on negative compared to neutral PM cues (*p* = 1.000). Thus there was a positive enhancement effect but no negative enhancement when comparing PM performance on negative valence cues with neutral cues.

**Table 2:** **Mean (M) and standard deviation (SD) for PM accuracy and PM accuracy at the recognition task for PD patients and Controls**.

Group	Emotional valence	PM (accuracy) *M (SD)*	PM content recognised *M (SD)*
PD patients	Positive	0.78 (0.22)	0.88 (0.17)
	Negative	0.69 (0.30)	0.75 (0.23)
	Neutral	0.64 (0.29)	0.71 (0.23)
Control group	Positive	0.93 (0.11)	0.95 (0.10)
	Negative	0.75 (0.20)	0.89 (0.16)
	Neutral	0.84 (0.19)	0.86 (0.22)

**Figure 2:**
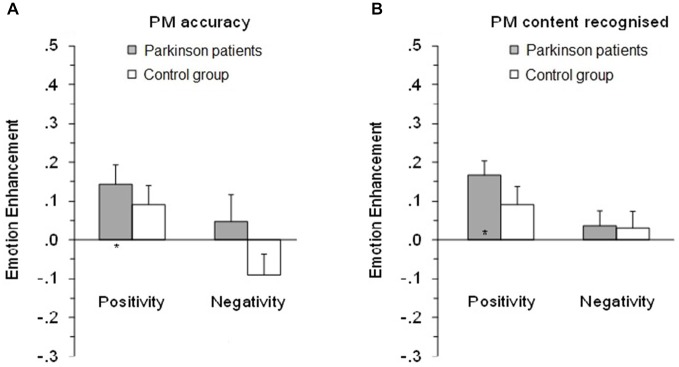
**Mean emotional enhancement/impairment index on the prospective memory (PM) tasks for PM accuracy (A) and PM task content recognized (B)**. Positivity index, proportion correct on positive minus neutral PM tasks. Negativity index, proportion correct on negative minus neutral PM tasks. *Enhancement/impairment index significantly different to zero. Error bars depict standard error of the mean.

The positivity enhancement was greater than zero for PD patients, *t*_(20)_ = 2.83, *p* = 0.010, and for controls the results were in the same direction but the greater positivity enhancement compared to zero only approached significance, *t*_(24)_ = 1.81, *p* = 0.083 (see Figure 2A, left side). Further analysis confirmed that the negative enhancement for PD patients *t*_(20)_ = 0.698, *p* = 0.493 and controls *t*_(24)_ = 1.67, *p* = 0.107 did not differ from zero.

### Recognition Test of PM Task Content

Participants’ performance on recognition test is reported in Figure 1B and Table 2. Group and emotional valence did not interact (*p* = 0.326, ${\text{η}}_{p}^{2}$ = 0.03), but there was a main effect of group, *F*_(1,44)_ = 6.70, *p* = 0.013, ${\text{η}}_{p}^{2}$ = 0.13, with PD patients (*M* = 0.78, *SD* = 0.22) less accurate than controls (*M* = 0.90, *SD* = 0.17). There was also a main effect of emotional valence, *F*_(2,88)_ = 11.07, *p* < 0.001, ${\text{η}}_{p}^{2}$ = 0.20. All participants were more accurate on positive PM cues (*M* = 0.92, *SD* = 0.14) than both negative (*p* = 0.001, *M* = 0.82, *SD* = 0.21) and neutral (*p* < 0.001, *M* = 0.79, *SD* = 0.23), but participants did not differ on negative compared to neutral PM cues (*p* = 0.845). Thus there was a positive enhancement effect but no negative enhancement or impairment when comparing PM performance on emotional valence cues with neutral cues.

Analysis of responses to distractors showed that both groups were at ceiling with respect to accuracy on rejecting distractors: PD patients (*M* = 0.98, *SD* = 0.04) and control group (*M* = 1.00, *SD* < 0.001). Indeed, there were only six and one exemplars of false alarms for the PD and control groups respectively. Thus control participants virtually always and Parkinson’s group nearly always selected “not required” as the cue for distracters.

As with the accuracy on PM tasks, to clarify the impact of emotional valence on the retrospective memory test, positivity and negative enhancement/impairment indices were analyzed (see Figure 2B, right side). The positive enhancement was greater than zero for PD patients, *t*_(20)_ = 4.64, *p* < 0.001, and for controls the results were in the same direction, but the greater positive enhancement compared to zero only approached significance, *t*_(25)_ = 1.89, *p* = 0.071. The negative enhancement (or impairment) did not differ compared to zero for both the participants with Parkinson’s, *t*_(20)_ = 0.90, *p* = 0.379, and for control, *t*_(24)_ = 0.68, *p* = 0.503.

### Neuropsychological Assessment

Table 3 reports mean, SD, *t*-test and Cohen’s d for neuropsychological assessment. The results suggested that PD patients were less accurate at the CPM task and recalled less word in semantic fluency task than controls. Moreover, in the delayed recall condition PD patients recalled fewer words in the word list task and less information in the prose recall task than controls. No differences were observed between groups in the TMT, both in time of performing Part A or Part B or when performance was analyzed in term of TMT Ratio score.

**Table 3:** **Descriptive and inferential statistics for the neuropsychological tasks included in the study**.

	PD patients *M (SD)*	Control group *M (SD)*	*t*	*d*
**CPM**	24.53 (5.69)	27.45 (3.27)	2.05*	0.62
**Semantic Fluency**	36.22 (7.89)	42 (6.79)	2.57*	0.78
**TMT**
Part A	84 (52)	73 (28)	0.93	0.27
Part B	130 (56)	147 (54)	0.99	0.31
B–A	53 (49)	74 (53)	1.29	0.41
**Word list**
Immediate recall	33.50 (7.72)	34.36 (8.57)	0.35	0.10
Delayed recall	6.85 (2.85)	8.68 (2.46)	2.31*	0.68
**Prose recall**
Immediate recall	13.37 (5.34)	15.36 (4.58)	1.33	0.40
Delayed recall	14.74 (5.11)	18.56 (4.77)	2.55*	0.77

Mean (M) and standard deviation (*SD)* of the PD patients and Controls; *t*-test values and effect size indices (Cohen’s *d*) are also indicated. Note: CPM, Raven’s Coloured Progressive Matrices; TMT, Trial Making Test in part A and B and difference in reaction time between Part B and Part A (B – A); Word list, World list recall task in immediate recall and delayed recall conditions; Prose recall, Prose recall tasks in the immediate recall and delayed recall conditions. *p < 0.05. Cohen’s d: Cohen (1988) defines effect sizes of 0.2 as small, 0.5 as medium, and 0.8 as large.

### Correlation Analyses

Correlation analyses were also conducted separately on PD patients and controls between PM accuracy (regardless of valence) and accuracy at the recognition task (regardless of valence). Significant correlations were observed in both PD patients (*r* = 0.66, *p* < 0.001) and controls (*r* = 0.44, *p* < 0.001).

Correlations were also conducted with neuropsychological tasks. In the case of PD patients, significant correlations were observed between MMSE (*r* = 0.34, *p* < 0.05), Semantic fluency (*r* = 0.35, *p* < 0.05) and PM accuracy; also significant correlation was observed between prose recall (delayed recall) and accuracy at the recognition task (*r* = 0.39, *p* < 0.05). In the case of controls, significant correlations were observed between Word list recall and Prose recall (both on delayed recall condition) and accuracy at the recognition task (*r* = 0.35, *p* < 0.05 and *r* = 0.48, *p* < 0.001, respectively).

Moreover, correlations were also conducted, in PD patients, with clinical indices (NPI, UPDRS and disease duration). Significant correlation were observed between NPI^2^ and PM accuracy (*r* = −0.50, *p* < 0.05) and between UPDRS and accuracy at the recognition task (*r* = 0.62, *p* < 0.001).

## Discussion

The purpose of the present study was to investigate PM performance in PD patients and healthy controls. Interestingly, the present study also examined the differences between groups in relation to the beneficial effects of emotional PM cues on PM performance. To this end, we used the Virtual Week task, a computer based task that simulates daily life activities and the effects of emotional cues on PM performance in individuals with PD and healthy participants were also compared. Finally, we investigated the retrospective memory component of PM by including a recognition task at the end of each virtual day.

Previous studies showed PM dysfunctions in PD patients in both time-based (Costa et al., 2008a, b) and event-based tasks (Katai et al., 2003; Kliegel et al., 2005; Whittington et al., 2006). The results of this study confirmed and extended previous findings in relation to event-based PM tasks and showed that PM performance in PD patients was significantly lower than that of the control group.

Typically, on event-based PM tasks, participants are instructed to perform a specific action when presented with a cue that is embedded in an ongoing activity. Event-based PM tasks are generally considered less cognitively demanding than time-based PM tasks because event-based tasks are triggered by an external cue, whereas time-based tasks involve self-initiated processes to monitor the time (McDaniel and Einstein, 1993; McDaniel et al., 1999; McFarland and Glisky, 2009; Mioni et al., 2015). However, even on event-based tasks that are considered relatively low in cognitive demands, our study found PD patients were less accurate than controls.

It is important to note that the event-based tasks used in the present study were different every virtual day and simulated the type of *irregular* activities that are new every day. The irregular activities required more cognitive resources and demanded more retrospective memory abilities than regular activities that are learned to criterion at the beginning of the game and repeated before starting each virtual day (Rose et al., 2010). The number of different intentions within a PM task (single vs. multiple; McDaniel and Einstein, 2000; Henry et al., 2007; Kliegel et al., 2011) or the complexity of their content (irregular vs. regular PM tasks, Rose et al., 2010; Foster et al., 2013) is likely to influence the amount of cognitive resources required to effectively encode and retrieve the PM intentions and thus may affect the performance in PM task.

PD patients often present executive dysfunction, even in the early stages of the disease (McKinlay et al., 2010). Disruption of fronto-striatal circuitry due to the depletion of dopamine in the basal ganglia and prefrontal cortex (PFC) is hypothesized as one of the underlying cause of PM difficulties (Middleton and Strick, 2000).

Although some studies have reported that retrospective problems do not interfere with PM performance in PD, very simple paradigms were used in those studies in which participants were asked to recall the instructions of the PM task (Katai et al., 2003; Kliegel et al., 2005; Costa et al., 2008b) or included recognition tasks not related to the PM tasks (Whittington et al., 2006). Therefore, previous studies did not adequately challenge the retrospective memory processes involved in PM. Interestingly, in the present study we included a recognition task at the end of each virtual day in which participants were asked to identify the PM activities required and also to indicate when the activity was required. The recognition task not only allowed us to investigate the retrospective memory component of PM actions, but it was also highly linked with the content of the PM tasks. If PD patients failed to execute the PM action during the virtual day we can test the recognition of that specific action during the recognition task and disentangle between prospective or retrospective memory dysfunction in PM tasks. The results for the recognition task indicate that lower PM performance observed in PD patients can also be caused by dysfunction in the retrospective memory component of PM process. Irregular tasks in Virtual Week are thought to impose high demands on retrospective memory processes. PD patients recalled less PM activities and made more errors (false alarm) during the recognition task. Taken together, these results suggest that the retrospective memory processes involved in PM can be disrupted by PD.

Further support for cognitive impairment being one of the possible causes of PM dysfunction in PD patients, comes from the significant correlations observed between measures of executive functions and PM accuracy and accuracy during the recognition task. In particular, in the case of PM accuracy, significant correlations were observed with indices of cognitive efficiency (MMSE) and executive functions (Semantic fluency); whereas in the case of the recognition task, significant correlation was observed with an index of retrospective memory (delayed recall in the prose recall task). The results confirmed previous findings highlighting the involvement of executive functions in PM performance (Kliegel et al., 2005; Foster et al., 2009; Raskin et al., 2011; Pirogovsky et al., 2012) and further support the notion of two distinct processes underlying PM performance. The retrospective component seems to be similar to the ability that is evaluated by retrospective memory tasks (Einstein and McDaniel, 1990, 1996) whereas, the PM component relies on executive functions and working memory (Kliegel et al., 2005; Costa et al., 2008a; Foster et al., 2009; Raskin et al., 2011).

The correlations of clinical indices are also of great interest in relation to the study of PM performance in PD patients. Our study showed that patients with more severe PD were also less accurate on both PM accuracy and accuracy at the recognition task.

The present study also addressed the question of emotionally related improvement in PM performance and this is the first study that investigated this issue in PD patients. We predicted that PM actions with emotional valence would be better performed compared to PM actions with neutral valence; in particular we expected a greater enhancement of stimuli with positive emotional valence (Kliegel and Jäger, 2006; Murphy and Isaacowitz, 2008; Clark-Foos et al., 2009; Altgassen et al., 2010; Rendell et al., 2011; Schnitzspahn et al., 2012). The data confirmed our prediction and showed better PM performance when the PM cue had a positive valence compared to both negative and neutral. However, the effect of emotional valence did not attenuate the PM dysfunction in PD patients relative to control, as PD patients performed worse than the control group independently of the emotional valence of the PM cue. Consistent, with previous studies conducted with older adults (Rendell et al., 2011) and clinical populations (Altgassen et al., 2011; Rendell et al., 2012) positive PM tasks were more likely to be performed than negative and neutral tasks.

We also calculated positivity and negativity effects (Murphy and Isaacowitz, 2008) by analyzing the difference between positive and neutral, and between negative and neutral PM tasks. This measure provides an indication of the size of any emotional enhancement or impairment effect, in a way that accounts to some degree for group differences in overall PM performance. This analysis revealed the different effects of emotional valence in PD patients and controls. A significant positive enhancement effect was found in PD patients, where they were better than the control group at remembering to perform tasks with positive emotional content relative to the neutral tasks.

Previous studies conducted with healthy older adults consistently showed the emotional effects on PM performance but the direction of these effects were diverse. Altgassen et al. (2010) and May et al. (2015) found a benefit in both young and older participants with emotional stimuli (positive and negative) compared to neutral, whereas, Ballhausen et al. (2015; Experiment 2) showed a reduction in PM performance when both positive and negative cues were presented. Our results are consistent with Rendell et al. (2011) and Schnitzspahn et al. (2012) who found enhanced PM performance for positive but not negative emotional PM cues.

It is possible that in forming and carrying out PM intentions, positive activities receive higher priority than negative intentions. This hypothesis fits our everyday experience; pleasant activities are more likely to be executed compared to unpleasant activities that are postponed and/or “forgotten”. More specific interpretation can be given considering the specificity of PD population.

A large number of different structures are involved in recognizing emotional stimuli: the occipitotemporal cortices, amygdala, orbitofrontal cortex, basal ganglia, and right parietal cortices, among others, in particular the dopaminergic system is believed to be critical in the emotion recognition process (Adolphs, 2002). Considering that most of these areas are compromised in PD patients, a consistent number of studies have shown emotion recognition dysfunction in PD patients. Although, most of the studies on emotional recognition in PD patients have been conducted with facial or vocal stimuli (Gray and Tickle-Degnen, 2010; Péron et al., 2012; Sotgiu and Rusconi, 2013), some of the main findings can apply to our results. In particular, Gray and Tickle-Degnen (2010) found than PD patients performed more poorly when the stimuli to be recognized referred to negative emotions (e.g., sadness, disgust and anger) compared to positive stimuli (e.g., happiness). It is possible that the positive enhancement observed was also determined by the preserved ability to detect positive emotional stimuli. It is possible that positive emotional stimuli attracted more attentional resources and positive emotional cues were better encoded, this produced facilitation in retrieving and executing the PM actions.

A limitation in the present study is the difference in the level of education in younger and older participants. However, it might be noted that this discrepancy is representative of the Italian population. Italian older adults often present with lower education levels as the majority of older adults only completed primary school (Mioni et al., 2015).

In summary, our study showed that PD patients were less accurate than control group in PM tasks. Also PD patients were less accurate than control group at recognition tasks indicating that PM dysfunction observed in PD patients is partially caused by retrospective memory dysfunction. The correlation analyses also showed that executive dysfunctions are involved in PM performance, indicating that PD patients with lower cognitive resources were also less accurate. These results were also confirmed by the correlations observed with the indices of severity (UPDRS and NPI). Interestingly, our results also showed positive enhancement of PM performance on event-based PM tasks. The positive enhancement observed in PD patients can have interesting implications for rehabilitation programs. In fact, future studies should further investigate the effects of positive stimuli in PD patients in PM performance and manipulate the presentation of the emotional cue at the encoding or retrieval phase.

## Conflict of Interest Statement

The authors declare that the research was conducted in the absence of any commercial or financial relationships that could be construed as a potential conflict of interest.

## Footnotes

^1^Focal/non-focal PM cues refer to the link between PM cue and ongoing task. If performing the ongoing task requires focal processing of the PM cue, then the cue is sufficiently processed to enable involuntary (automatic) retrieval of the intended action. In other cases, if the ongoing task does not direct attention toward processing the features of the PM cue than the PM cue can be considered nonfocal.

^2^Lower score at NPI represents better performance.
